# Accurate prediction of protein–nucleic acid complexes using RoseTTAFoldNA

**DOI:** 10.1038/s41592-023-02086-5

**Published:** 2023-11-23

**Authors:** Minkyung Baek, Ryan McHugh, Ivan Anishchenko, Hanlun Jiang, David Baker, Frank DiMaio

**Affiliations:** 1 School of Biological Sciences, Seoul National University, Seoul, Republic of Korea; 2 Department of Biochemistry, University of Washington, Seattle, WA USA; 3 Institute for Protein Design, University of Washington, Seattle, WA USA; 4 Department of Electrical Engineering and Computer Sciences, University of California, Berkeley, CA USA; 5 Howard Hughes Medical Institute, University of Washington, Seattle, WA USA

**Keywords:** Machine learning, DNA-binding proteins, RNA-binding proteins

## Abstract

Protein–RNA and protein–DNA complexes play critical roles in biology. Despite considerable recent advances in protein structure prediction, the prediction of the structures of protein–nucleic acid complexes without homology to known complexes is a largely unsolved problem. Here we extend the RoseTTAFold machine learning protein-structure-prediction approach to additionally predict nucleic acid and protein–nucleic acid complexes. We develop a single trained network, RoseTTAFoldNA, that rapidly produces three-dimensional structure models with confidence estimates for protein–DNA and protein–RNA complexes. Here we show that confident predictions have considerably higher accuracy than current state-of-the-art methods. RoseTTAFoldNA should be broadly useful for modeling the structure of naturally occurring protein–nucleic acid complexes, and for designing sequence-specific RNA and DNA-binding proteins.

## Abstract (web summary)

RoseTTAFoldNA extends the RoseTTAFold2 platform to predict the structures of protein–DNA and protein–RNA complexes.

## Main

Current approaches for protein–nucleic acid complex structure prediction involve building models of the protein and nucleic acid (NA) components separately and then building up complexes using computational docking calculations^1–3^. For predicting protein components, machine learning-guided approaches like RoseTTAFold^4^ and AlphaFold^5^ are highly accurate, while RNA structure prediction has used a combination of Monte Carlo sampling approaches^6–9^ as well as deep learning methods^10,11^. Despite this progress in predicting individual components, the prediction of the structure of protein–nucleic acid complexes has lagged considerably behind the prediction of protein structures or RNA structures alone.

AlphaFold and RoseTTAFold take as input one or more aligned protein sequences, and successively transform this information in parallel one-dimensional (1D), two-dimensional (2D) and—in the case of RoseTTAFold—three-dimensional (3D) tracks, ultimately outputting three-dimensional protein structures. The 10 s to 100 s of millions of free parameters in these deep networks are learned by training on large sets of proteins of known structures from the Protein Data Bank (PDB). Both AlphaFold and RoseTTAFold can generate accurate models of not only protein monomers but also protein complexes, modeling folding and binding by successive transformations over hundreds of iterations. Given the overall similarities between protein folding and RNA folding, and between protein-protein binding and protein–nucleic acid binding, we reasoned that the concepts and techniques underlying AlphaFold and RoseTTAFold could be extended to the prediction of the structures of nucleic acids and protein–nucleic acid complexes from sequence information alone. We set out to generalize RoseTTAFold to model nucleic acids in addition to proteins, and to learn the many new parameters required for general protein–nucleic acid systems by training on the structures in the PDB. A major question at the outset was whether there were sufficient nucleic acid and protein–nucleic acid structures in the PDB to train an accurate and general model; key to the success of AlphaFold are the hundreds of thousands of protein structures in the PDB, but there are an order of magnitude fewer nucleic acid structures and complexes. The flexibility of nucleic acids relative to proteins could also make the prediction of the former more difficult.

Our new model, RoseTTAFoldNA, was trained using the same data as RoseTTAFold, augmented with all RNA, protein–RNA and protein–DNA complexes in the PDB. Using nucleic acid complexes published more recently than any training-set examples, we evaluate its ability to predict structures of protein–nucleic acid complexes without homologs. We also assess the model’s self-assessments of model accuracy, and compare our predictions to a combination of AlphaFold and computational protein–DNA docking.

## Results

**Fig. 1:**
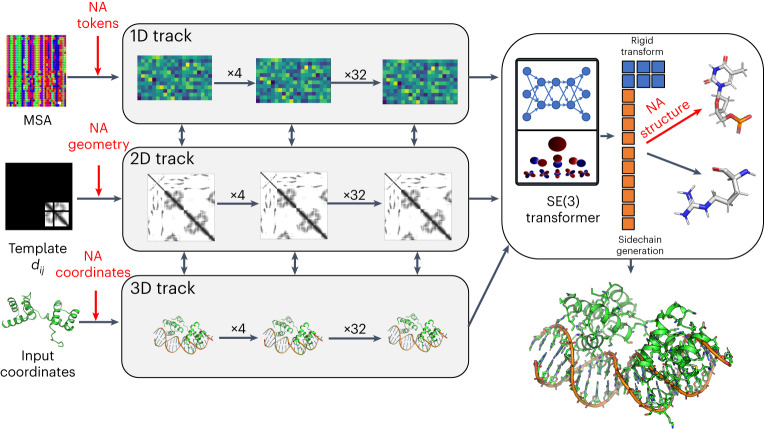
Overview of the architecture of RoseTTAFoldNA. The three-track architecture of RoseTTAFoldNA simultaneously updates sequence (1D), residue-pair (2D) and structural (3D) representations of protein–nucleic acid complexes. The areas in red highlight key changes necessary for the incorporation of nucleic acids: inputs to the 1D track include additional NA tokens, inputs to the 2D track represent template protein–NA and NA–NA distances (and orientations) and inputs to the 3D track represent template or recycled NA coordinates. Finally, the 3D track as well as the structure refinement module (upper right) can build all-atom nucleic acid models from a coordinate frame (representing the phosphate group) and a set of 10 torsion angles (six backbone, three ribose ring and one nucleoside). In this figure, d_ij_ are the template inter-residue distances, and SE(3) refers to the Special Euclidean Group in three dimensions.

The architecture of RoseTTAFoldNA (RFNA) is illustrated in Fig. 1. It is based on the three-track architecture of RoseTTAFold^4^, which simultaneously refines three representations of a biomolecular system: sequence (1D), residue-pair distances (2D) and cartesian coordinates (3D). In addition to several modifications to improve performance^12^, we extended all three tracks of the network to support nucleic acids in addition to proteins. The 1D track in RoseTTAFold has 22 tokens, corresponding to the 20 amino acids, a 21st ‘unknown’ amino acid or gap token and a 22nd mask token that enables protein design; to these, we added 10 additional tokens, corresponding to the four DNA nucleotides, the four RNA nucleotides, unknown DNA and unknown RNA. The 2D track in RoseTTAFold builds up a representation of the interactions between all pairs of amino acids in a protein or protein assembly; we generalized the 2D track to model interactions between nucleic acid bases and between bases and amino acids. The 3D track in RoseTTAFold represents the position and orientation of each amino acid in a frame defined by three backbone atoms (N, CA and C), and up to four chi angles to build up the sidechain. For RoseTTAFoldNA, we extended this to include representations of each nucleotide using a coordinate frame describing the position and orientation of the phosphate group (P, OP1 and OP2), and 10 torsion angles which enable the building up of all the atoms in the nucleotide. RoseTTAFoldNA consists of 36 of these three-track layers, followed by four additional structure refinement layers, with a total of 67 million parameters.

We trained this end-to-end protein–NA structure prediction network using a combination of protein monomers, protein complexes, RNA monomers, RNA dimers, protein–RNA complexes and protein–DNA complexes, with a 60/40 ratio of protein-only and NA-containing structures (Methods). Multichain assemblies other than the DNA double helix were broken into pairs of interacting chains. For each input structure or complex, sequence similarity searches were used to generate multiple sequence alignments (MSAs) of related protein and nucleic acid molecules. Network parameters were optimized by minimization of a loss function consisting of a generalization of the all atom Frame Aligned Point Error (FAPE) loss^5^ defined over all protein and nucleic acid atoms (Methods) together with additional contributions assessing the recovery of masked sequence segments, residue-residue (both amino acids and nucleotides) interaction geometry and error prediction accuracy. To try to compensate for the far smaller number of nucleic-acid-containing structures in the PDB (following sequence-similarity-based cluster to reduce redundancy, there are 1,632 RNA clusters and 1,556 protein–nucleic acid complex clusters compared to 26,128 all protein clusters), we also incorporated physical information in the form of Lennard-Jones and hydrogen-bonding energies^13^ as input features to the final refinement layers, and as part of the loss function during fine-tuning. During training, 10% of the clusters were withheld for model validation.

We trained the model using structures determined prior to May 2020, and used RNA and protein–NA structures solved since then as an additional independent validation set. For the validation set, complexes were not broken into interacting pairs and were processed entirely as full complexes. Paired MSAs were generated for complexes with multiple protein chains as described previously^14^. Due to GPU memory limitations, for the validation set only, we excluded complexes with more than 1,000 total amino acids and nucleotides, which resulted in a validation set containing 520 cases (98 clusters) with a single RNA chain, 224 complexes (116 clusters) with one protein molecule plus a single RNA chain (62/28 clusters) or DNA duplex (162/88 clusters), and 161 cases with more than one protein chain or more than a single RNA chain or DNA duplex.

### Predicting protein–NA complexes

**Fig. 2:**
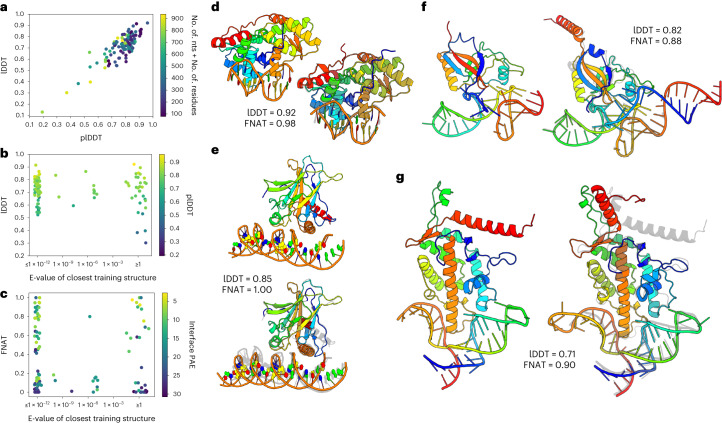
Protein–nucleic acid structure prediction. **a**–**c**, Summary of results on 32 protein–NA cluster representatives from the validation set and 84 protein–NA structures released since May 2020. **a**, Scatterplot of prediction accuracy (true lDDT to native structure) versus prediction confidence (lDDT predicted by the model) shows that the model correctly identifies inaccurate predictions. **b**, The model seems to generalize well, with no clear performance difference between structures with and without sequence homologs in the protein–NA training set. **c**, Scatterplot of native interface contacts recapitulated in the prediction (FNAT) versus sequence similarity to training data. A total of 35% of predictions are ranked ‘acceptable’ or better by CAPRI metrics, and 78% of those with high confidence (mean interface PAE < 10). **d**–**g**, Four examples of protein–NA complexes without homologs in the training set: the BpuJ1 endonuclease bound to a modified cognate DNA (**d**, PBD ID: 5hlt)^21^; tumor antigen p53 bound to cognate DNA with induced-fit sequence specificity (**e**, PDB ID: 3q05)^22^; SmpB bound to the tRNA-like domain of a transfer-messenger RNA (**f**, PDB ID: 1p6v)^23^; and a telomerase reverse transcriptase bound to the enzyme’s RNA component (**g**, PDB ID: 4o26)^24^. Source data

RoseTTAFoldNA results on 224 monomeric protein–NA complexes are summarized in Fig. 2, shown as 116 clusters. The predictions are reasonably accurate, with an average Local Distance Difference Test (lDDT) of 0.73 and 29% of models with lDDT > 0.8 (19% of clusters, Fig. 2a), and about 45% of models contain greater than half of the native contacts between protein and NA (fraction of native contacts, FNAT > 0.5, 35% of clusters, Fig. 2c). RoseTTAFoldNA, like RoseTTAFold and AlphaFold, outputs not only a predicted structure but also a predicted model confidence, and as expected the method correctly identifies which structure models are accurate. Although only 38% of the complexes (28% of clusters) are predicted with high confidence (mean interface predicted aligned error, PAE < 10), of those, 81% (78% of clusters) correctly model the protein–NA interface (‘acceptable’ or better by CAPRI metrics^15^). Over the 33 clusters with no detectable sequence similarity to training protein–NA structures, the accuracy is similar (average lDDT = 0.68 with 24% of models > 0.8 lDDT and 42% with FNAT > 0.5), and the model is still able to correctly identify accurate predictions—24% of predictions in this subset are predicted with high confidence, of which all eight have acceptable interfaces according to CAPRI metrics. Four predictions of structures with no sequence homologs in the training set are shown in Fig. 2d–g. These include the endonuclease BpuJ1, tumor antigen p53, SmpB bound to a tRNA-like RNA domain, and components of a telomerase reverse transcriptase. Inaccuracies in these predictions can be found in flexible terminal regions (Fig. 2e,g), a slight tilt of the DNA double helix relative to the interface (Fig. 2e) and slight deviations in RNA tertiary structure (Fig. 2f,g), but the interfaces are clearly correct.

**Extended Data Fig. 1:**
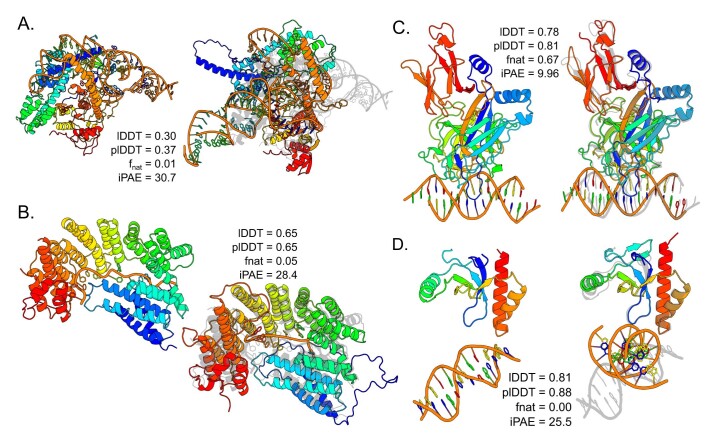
Failure modes of protein - nucleic acid structure prediction. (**a**–**d**) Comparisons of representative predictions showing common failure modes of predictions in cases with no training-set homologs. Left is the deposited model, and right is the prediction. (A) Example where the individual subunits predict with poor accuracy, resulting in an incorrect overall complex (pdb ID: 6XMF). Cases like this represent 50% of the examined failures and often result from very large or very small single-stranded nucleic acids (>100 or <20 nucleotides), large multi-domain proteins, or heavily distorted duplex DNAs. (B) Example where the subunits predict with reasonable accuracy and the relative orientation is correct but the details of the interface are wrong (pdb ID: 7A9X). Cases like this represent 20% of the examined failures, and can also result from small single-stranded nucleic acids or slight deviations in monomer structures. (C) Example where the subunits predict with high accuracy and the backbone-backbone binding mode is correct, but the interface is predicted at the wrong site on the DNA (pdb ID: 4J2X). Cases like this represent 10% of the examined failures. (D) Example where both subunits predict correctly but the relative orientation and interface are incorrect (pdb ID: 7LH9). Cases like this represent 20% of the examined failures, and can result from distorted or non-duplex DNA structures or slight deviations in monomer structures.

In cases where RoseTTAFoldNA fails to produce an accurate prediction, the most common cause is poor prediction of individual subunits, typically large multidomain proteins, large RNAs (>100 nt) and small single-stranded nucleic acids. When the subunit predictions are accurate, the most common failure mode is for the model to identify either the correct binding orientation or the correct interface residues, but not both. The remaining cases with completely incorrect interfaces often involve only glancing contacts or heavily distorted DNAs. It is possible that a different training schedule could reduce these errors, but more likely it is due to limited training data in these regimes. Extended Data Fig. 1 illustrates some examples.

**Fig. 3:**
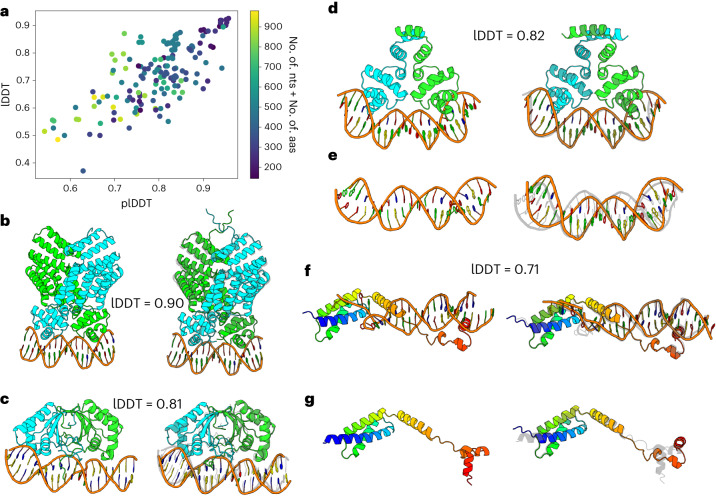
Modeling multichain protein–nucleic acid complexes. **a**, Scatterplot of predicted model accuracy versus actual model accuracy for 161 protein–NA complexes with multiple protein chains or multiple nucleic acid chains/duplexes shows that the model accurately estimates error. **b**–**d**,**f**, Examples of successful predictions without homologs in the training set, shown as the deposited model (left) and prediction (right). These include the viral chromatin anchor KSHV LANA (**c**, PDB ID: 4uzb)^25^, two dimeric helix-turn-helix transcription factors (**b**, PDB ID: 3u3w; panel D, PDB ID: 4jcy)^26,27^ and a replication origin unwinding complex (**f**, PDB ID: 3vw4)^28^. **e**,**g**, Example showing different predicted conformations of the same protein or DNA duplex alone (left) and with the other component (right), from the same complexes shown in **d** (**e**) and **f** (**g**). Source data

RoseTTAFoldNA prediction is not limited to complexes with only a single protein subunit. Figure 3 summarizes the performance of RoseTTAFoldNA on 161 multisubunit protein–NA complexes, most of which are homodimeric proteins bound to nucleic acid duplexes. The performance is similar to that for monomeric protein–nucleic acid complexes, with an average lDDT = 0.72 with 30% of cases >0.8 lDDT, and good agreement between confidence and accuracy (Fig. 3a). Three examples are illustrated in Fig. 3b–d, showing the ability of the model to predict complex structure as well as the ‘bending’ of DNA induced by protein binding (Fig. 3e). Figure 3f,g shows another example where the relative positioning of protein domains is only made by copredicting these complexes. Such effects would not be possible to predict by approaches that first generate models of the independent components and then rigidly dock them.

### Predicting RNA complexes

**Extended Data Fig. 2:**
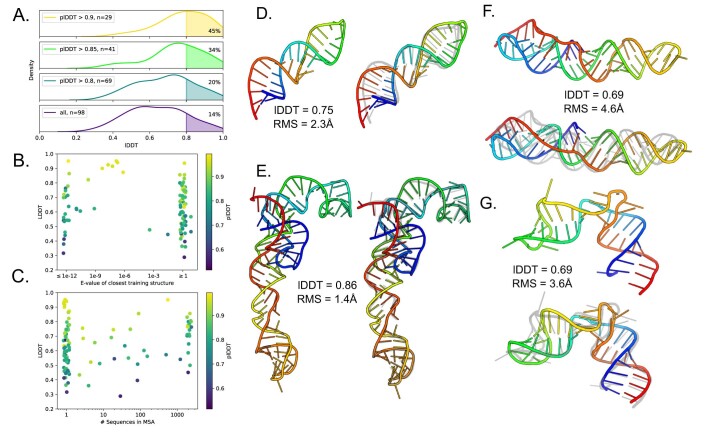
RNA structure prediction. (**a**–**c**) Summary of results on 55 RNA cluster representatives from the validation set and 43 RNA structures released since May 2020. (A) Model accuracy increases at higher confidence levels. The overall average lDDT is 0.64, and the average lDDT for very high confidence predictions (predicted lDDT > 0.9) is 0.78. (B) The model shows little to no performance decrease for RNA molecules with no sequence homologs in the training set. (C) Average accuracy improves as the number of sequences in the MSA increases, but many single-sequence examples are accurately predicted. (**d**–**f**) Four example predictions of RNA models with no detectable sequence homologs in the training set, two of which also have no detectable structural homology according to PDB structure similarity search. (D) a simple hairpin RNA fragment from the 16S rRNA (PDB id: 1i6u), (E) the 5S rRNA from a full ribosome structure (PDB id: 3jai), (F) the SARS-CoV-2 frameshifting pseudoknot RNA (PDB id: 7lyj), and (**g**) a 49-nt mRNA fragment, solved bound to a ribosomal protein (PDB id: 1u63). Source data

Finally, RoseTTAFoldNA performance on RNA structures alone are summarized in Extended Data Fig. 2. Most predictions are reasonably accurate: the average lDDT is 0.73, with 48% of models (but only 14% of clusters) predicted with lDDT > 0.8 (Extended Data Fig. 2a). 62% of cases (30% of clusters) are predicted with very high confidence (predicted lDDT, plDDT > 0.9), for which the average lDDT is 0.81 and 77% of models (45% of clusters) have lDDT > 0.8. Even for cases with no homologs of known structure or small numbers of sequence relatives (shallow MSAs), confidently predicted models are generally quite accurate (colourbar, Extended Data Fig. 2b,c) and the network is capable of predicting structures without detectable homologs in the training dataset (Extended Data Fig. 2d–g).

## Discussion

At the outset of this work, it was not clear that there were enough protein–nucleic acid structures in the PDB to enable robust training of a deep learning-based predictor with atomic accuracy—the training data used for nucleic acid prediction is only one tenth the size of the dataset used for protein structure prediction. Our results show, however, that this data is sufficient in many cases for de novo structure modeling, with accurate modeling of protein–NA interfaces without shared MSA information or homologs of known structure in about 31% of cases. Prospective and blind tests will be important for further critical evaluation of the method. Along these lines, we made predictions for CASP15 RNA targets during CASP with an earlier version of RoseTTAFoldNA.

**Extended Data Fig. 3:**
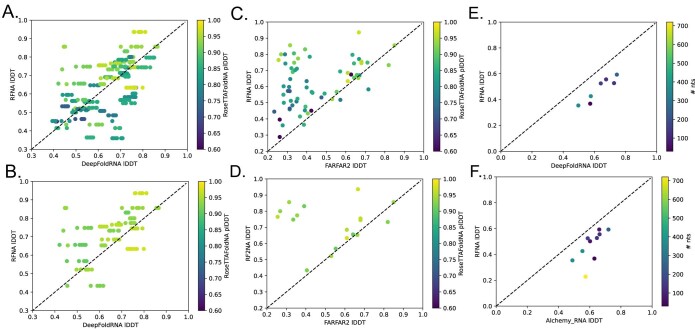
Comparing RoseTTAFoldNA to other methods for RNA prediction. (**a**) Scatterplot of predicted accuracy for RoseTTAFoldNA versus DeepFoldRNA, a recent machine learning method for RNA structure prediction^15^. RoseTTAFoldNA has similar performance to DeepFoldRNA, with average lDDTs of 0.64 and 0.64 respectively. (**b**) RoseTTAFold outperforms DeepFoldRNA if only RoseTTAFold’s high-confidence predictions (predicted lDDT > 0.9) are considered, which have an average lDDT of 0.72. (**c**) Scatterplot comparing RoseTTAFoldNA to FARFAR2, a Rosetta-based fragment assembly method for RNA structure prediction^4^. FARFAR2 results show the best model by Rosetta energy, of 100 predictions or the number completed in 24 CPU-hours. RoseTTAFoldNA consistently and dramatically outperforms FARFAR2’s top-ranked models, which have an average lDDT of 0.44. (**d**) The performance gap is similar when only considering RoseTTAFoldNA confident predictions. (**e**, **f**) Comparisons between RoseTTAFoldNA and other machine learning methods on the CASP15 RNA targets (using model 1 of each method). RFNA performs somewhat worse than DeepFoldRNA and significantly worse than AIchemy_RNA, the leading machine learning method from the competition. Source data

**Extended Data Fig. 4:**
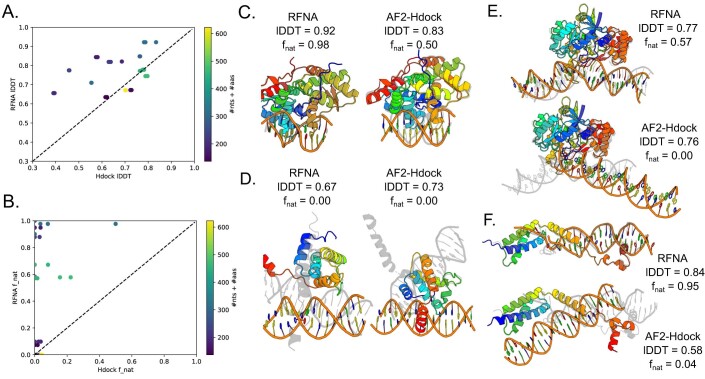
Comparing RoseTTAFoldNA to docking of monomer predictions. (**a**) Scatterplot comparing overall structure accuracy of RFNA versus the top 3 ranked docks from Hdock template-free docking of predicted protein monomers with predicted RNAs or B-form DNAs. (**b**) Scatterplot comparing interface contact recovery of RFNA predictions versus the top 3 models from the docking calculations. (**c–f**) Example predictions from both methods shown with the deposited model shown as a light gray silhouette. (C) Example where both RFNA and Hdock’s third-ranked dock successfully recover the correct interface (PDB id: 5HLT). Example where neither RFNA nor Hdock identify the correct orientation of protein and DNA (PDB id: 7V9F) []. Note that both RFNA and AF2 predict the protein in a different conformation than the one found in the deposited model, making complex formation difficult. (E) Example where RFNA predicts the correct complex while Hdock does not reproduce the interface (PDB id: 7K33). Note that the distorted DNA structure would be difficult to model using any traditional methods. (F) Another example where RFNA is successful but docking is not, again with a distorted DNA structure that is difficult to predict (PDB id: 3VW4). Source data

Comparison of RoseTTAFoldNA to current state-of-the-art methods is more difficult than the case for the deep learning methods AlphaFold and RoseTTAFold which focused on the much more well studied protein structure prediction problem. There has been recent work on RNA structure prediction; Extended Data Fig. 3 shows the performance of this network compared to the traditional sampling-based FARFAR2 method^4^ and the deep learning-based DeepFoldRNA method^15^. FARFAR2 and DeepFoldRNA top-ranked models have average lDDTs of 0.44 and 0.64, respectively, compared to 0.62 for RoseTTAFoldNA. On the CASP15 RNA targets, we perform worse than the leading machine learning methods DeepFoldRNA and AIchemy—but most of the targets are quite large and several are synthetic RNA origamis with no MSAs^16^. For protein structure prediction, we see performance in-line with AlphaFold, with an average TM-score of 0.87 for RFNA versus 0.88 for AlphaFold (comparing AlphaFold ‘model 1’ and using the same MSA for both AlphaFold and RFNA). While the performance of individual modalities is not an advancement over state-of-the-art, the strength of RoseTTAFoldNA is in the prediction of protein–nucleic acid complexes. Here, comparisons are more difficult, as there are no equivalent deep learning-based methods, and even sampling-based methods have focused more on bespoke solutions to a specific problem rather than general methods. While automated methods are available for predicting individual protein, RNA, and DNA components and for energy-based docking of macromolecules, we find that this alternative workflow has very poor accuracy, finding the correct complex within the top three models in only 1 of 14 test cases (see Methods for details on our workflow and Extended Data Fig. 4 for detailed results). Hence, while the accuracy of RoseTTAFoldNA on protein–nucleic acid complexes is considerably lower than that of AlphaFold on protein structures, it represents a notable improvement in the state-of-the-art.

**Extended Data Fig. 5:**
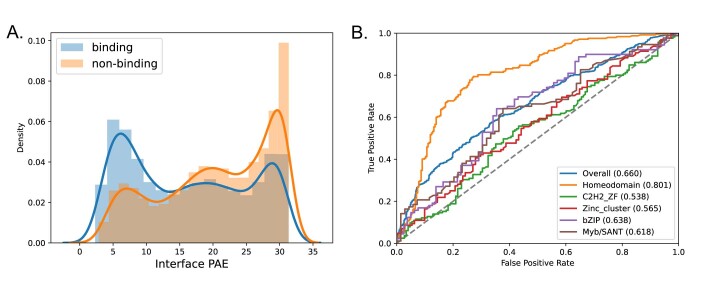
Using RoseTTAFoldNA to distinguish binding and non-binding DNA sequences for transcription factors. (**a**) Plot showing distribution of the model’s interface confidence estimate for proteins predicted with binding and non-binding DNA sequences. (**b**) ROC curve showing how well the binding DNA sequences can be selected from the pool of binding and nonbinding sequences based on the model’s predicted accuracy scores. Curves are shown for all proteins and for the five most common protein families in the dataset. Source data

Further increases in accuracy might come from a larger, more expressive network; we used a smaller network than that of RoseTTAFold, with ∼67 M parameters and 36 total layers. Use of high-confidence predicted structures as additional training examples (made more difficult by subsampling MSAs) should further increase model accuracy^10^; for this purpose there are databases of structured RNAs^17,18^ and DNA-binding profiles for thousands of proteins^19,20^, and the latter should be useful for training a model fine-tuned for DNA specificity as well (see Methods and Extended Data Fig. 5 for RoseTTAFoldNA performance on DNA-binding specificity prediction). Deep learning-guided structure prediction of proteins has opened up new avenues of research; we hope that RoseTTAFoldNA does the same for protein–NA interactions and complexes. To this end, we have made the method freely available.

## Methods

### Training and validation data processing

The protein and protein complex data used in training was identical to that used in training RoseTTAFold2. Additional data from RNA and protein–nucleic acid complexes was added to this. To construct this dataset, all PDBs solved by nuclear magnetic resonance, crystallography or cryo-electron microscopy at better than 4.5 Å resolution were collected. A dataset was constructed considering all PDB structures published at or before 30 April 2020, and collecting:

- All RNA single chains and all RNA duplexes. A duplex was defined by looking for pairs of RNA chains making at least 10 hydrogen bonds.
- All interacting protein–nucleic acid pairs. Interacting pairs were defined by counting the number of 7 Å contacts between protein Cαs and any (non-hydrogen) nucleic acid atom; if there were more than 16 such contacts, the pair was considered interacting. Nucleic acid duplexes were included if the DNA or RNA chains made at least 10 hydrogen bonds.

For modeling, the full-length sequence was used. All non-standard bases/amino acids were converted into a backbone-only ‘unknown’ residue type. The dataset size was 7,396 RNA chains and 23,583 complexes. These were then clustered using a 1 × 10^−3^ hhblits^29^ E-value for proteins and 80% sequence identity for RNA molecules, yielding 1,632 non-redundant RNA clusters and 1,556 non-redundant protein–NA clusters. These clusters were then split into training and validation sets, with clusters chosen for the training set; an example which contained any member (NA or protein) of a validation set cluster was assigned to the validation set. This led to 199 protein–NA clusters and 116 RNA clusters in the validation set.

Multiple sequence alignments (MSAs) were then created for all protein and RNA sequences in the training and validation set. Protein MSAs were generated in the same way as RoseTTAFold^12^, using hhblits at successive E-value cutoffs (1 × 10^−30^, 1 × 10^−10^, 1 × 10^−6^ and 1 × 10^−3^), stopping when the MSA contains more than 10,000 unique sequences with >50% coverage. RNA MSAs were generated using a pared-down version of rMSA (https://github.com/pylelab/rMSA) that removes secondary structure predictions: sequences were searched using blastn^30^ over three databases (RNAcentral^17^, rfam^18^ and nt) to first identify hits, then using nhmmer^31^ to rerank hits. We again use successive E-value cutoffs (1 × 10^−8^, 1 × 10^−7^, 1 × 10^−6^, 1 × 10^−3^, 1 × 10^−2^ and 1 × 10^−1^), stopping when the MSA contains more than 10,000 unique sequences with >50% coverage.

Finally, to improve generalizability of protein–DNA interactions we added a few ways of ‘randomizing’ inputs during training. As many crystal structures of protein–DNA complexes involve short DNA chains with the binding motif in the middle, initial versions of the model had a strong preference to binding in the middle of any provided sequence. To deal with this, we added a random padding of 0–6 nucleotides to both ends of all native structures: (1) containing double-stranded DNA and (2) making at least three base-specific contacts (using a cutoff distance of 3.4 Å). This yielded 580 protein–DNA complexes. These added residues were not included in loss calculations, but were present in the predicted structures. Additionally, we also performed negative training for these same 580 complexes; all DNA bases forming base-specific contacts to the bound protein were randomly mutated (maintaining Watson–Crick base pairing), and the model was trained to move the protein and DNA far apart (by favouring the 6-dimensional ‘distogram’ loss to place all its probability mass in the final bin).

### Test set data processing

For an independent test set, we took all structures published to the PDB 1 May 2020 or later. Selection criteria and preprocessing was the same as for the training and validation data with two exceptions: (1) only complexes fewer than 1,000 residues plus nucleotides in length were considered and (2) for complexes containing more than one unique protein chains, paired MSAs were created by merging sequences from the same organism into a single combined sequence (following prior work^14^). This gave us 91 complexes with one protein molecule plus a single RNA chain or DNA duplex, 43 cases with a single RNA chain and 106 cases with more than one protein chain or more than a single RNA chain or DNA duplex.

### All atom generation for nucleotides

Following AlphaFold’s treatment of amino acids, when predicting structure, the model represents each nucleotide as a rigid frame (with a rotation and translation) and a set of internal torsion angles. For nucleic acids this frame corresponds to the orientation of the phosphate group (O–P–O), in the same way that N–Cα–C is used as an amino acid frame. A set of ten torsions describe the placement of all sidechain atoms, representing the rotatable bonds in the nucleotide: six backbone (*α*, *β*, *γ*, *δ*, *ϵ* and *ζ*), one sidechain (*χ*) and three additional angles controlling ribose ‘pucker’ (*ν*_0_, *ν*_1_ and *ν*_2_). When all atom models are generated as part of the loss calculation, they are kinematically folded outward from the phosphate group following the chain of torsions connecting them.

### Loss functions

The model was trained using a loss function similar to RoseTTAFold, where we take the weighted sum:

$$\text{loss}={w}_{\rm{seq}}\times \text{seq}+{w}_{\rm{6D}}\times 6\text {D}+{w}_{\rm{str}}\times \text{str}+{w}_{\rm{tors}}\times \text{tors}+{w}_{\rm{err}}\times \text{err}$$

Above, seq is the masked amino acid recovery loss (no masking is applied to nucleotide sequences); 6D is the six-dimensional ‘distogram’ loss^32^; str is the structure loss, consisting of the average backbone FAPE loss^5^ over all 40 structure layers of the network plus the all atom FAPE loss for the final model; tors is the torsion prediction loss averaged over the 40 structure layers; err is the loss in pLDDT prediction; and the *w* terms are the weights on individual components in the loss function.

FAPE loss is extended to nucleic acids in a straightforward manner from how it is implemented for amino acids. For backbone FAPE loss, the phosphate group (O–P–O) in the nucleic acid backbone is treated as the nucleotides ‘frame.’ For nucleic acid all atom FAPE loss, three-atom frames are constructed corresponding to each of the ten ‘rotatable torsions’ (see above), where the frame consists of the two bonded atoms defining the torsion plus an additional bonded atom, closer to the phosphate group in the bond graph. The cross product of these ten frames with all atoms is used to calculate FAPE loss.

Following training with the above loss function, an additional ‘fine-tuning’ phase is carried out, where additional energy terms are added to the loss function enforcing reasonable model geometry:

$$\begin{array}{l}{\text{loss}}_{\text{finetune}}=\text{loss}+{w}_{\text{LJ}}\times \text{LJ}+{w}_{\text{hbond}}\times \text{hbond}\\\qquad\qquad\quad+{w}_{\text{geom}}\times \text{geom}+{w}_{\text{pairerr}}\times \text{pairerr}\end{array}$$

Above, LJ and hbond are the Lennard-Jones and hydrogen bond energies of the final structure (normalized by the number of atoms), using a reimplementation of the corresponding Rosetta energy terms^13^; geom is a term that enforces ideal bond lengths and bond angles around the peptide or phosphodiester bond connecting residues/nucleotides; and pairerr is a predicted residue-pair error^5^. The functional form of the geom term is identical to that of RoseTTAFold2, a linear penalty with a ‘flat bottom’ ±3°/0.02 Å from the ideal values.

### Model training

The network was trained in two stages, an initial training period, and a fine-tuning period. In both, input structures were divided into five pools: (1) protein structures, (2) ‘distilled’ protein structures (consisting of high-confidence AlphaFold predictions), (3) protein complexes, (4) protein–NA complexes and (5) RNA structures. Training sampled from each of these pools with equal probability (though later in training protein–NA frequency was increased to 25% and RNA frequency lowered to 15%). For both pools containing ‘complexes,’ an equal number of positive and negative examples were used in training. Negative examples consist of nonbinding proteins or protein–NA pairs; the structure loss only penalizes each component individually, and the 6D loss favors placing negative binding examples far apart.

Examples larger than 256 residues/nucleotides in length were ‘cropped’ to 256 residues in length. For protein-only data these crops were continuous sequences; for nucleic acids and nucleic acid–protein complexes the cropping was a bit more complex. A graph was constructed where sequential residues/nucleotides had edges with weight 1, Watson–Crick base-paired nucleotides had weight 0 and protein–NA bases closer than 12 Å (Cα to P) had a weight of 0. In negative cases, a single random protein–NA edge was given weight 0. Then minimum-weight graph traversal starting from a randomly chosen protein–NA edge was used to crop the model down to 256 residues/nucleotides. For RNA-only models the same strategy was used, though the starting point was a random nucleotide.

Training was carried out in parallel on 64 GPUs. A batch size of 64 was used throughout training with a learning rate of 0.001, decaying every 5,000 steps. The following weights were used: *w*_seq_ = 3.0, *w*_6d_ = 1.0, *w*_str_ = 10.0, *w*_tors_ = 10.0 and *w*_err_ = 0.1. The Adam optimizer was used, with L2 regularization (coeff = 0.01).

Following ∼1 × 10^5^ optimization steps, fine-tuning training was carried out. Here we increase crop size to 384 and effective batch size to 128, and reduce learning rate to 5× 10^−4^. We used additional loss terms with weights *w*_geom_ = 0.1, *w*_LJ_ = 0.02, *w*_hbond_ = 0.05 and *w*_pairerr_ = 0.1, and optimized for an additional 30,000 minimization steps. All told, training took approximately 4 weeks.

### Protein–nucleic acid docking

From the protein–nucleic acid complexes with no homologs in RFNA’s training set, we selected eight protein–DNA complexes and six protein–RNA complexes to use as test cases for docking. Protein monomer structures were predicted with AlphaFold^5^, using the same MSAs generated for RFNA predictions and choosing the prediction with the highest average predicted lDDT from models 1–5. RNA components were predicted using DeepFoldRNA following the default instructions. DNA duplexes were generated as B-form helices using x3DNA^33^. Docking was performed using the Hdock web server^34^, using only template-free docking to avoid fitting directly to the original deposited model. Structure and interface accuracy of the top three docks were evaluated as for RFNA. We acknowledge that a more careful DNA modeling and docking workflow could produce more accurate models, but similar could be said for RFNA.

### Binding and nonbinding DNA sequence dataset

We obtained experimental data of transcription factors’ DNA-binding profiles from the Cis-BP database^19^. We used 1,509 proteins for which the protein sequences of the experimental constructs and DNA 8mer E-scores were available. From the 8mer E-scores for each protein, we chose the top three most enriched DNA sequences as ‘binding’ and three random negatively enriched DNA sequences as ‘nonbinding’. We predicted the proteins and DNAs together using RFNA and evaluated the model based on the average PAE across the interface.

### Reporting summary

Further information on research design is available in the Nature Portfolio Reporting Summary linked to this article.

## Online content

Any methods, additional references, Nature Portfolio reporting summaries, source data, extended data, supplementary information, acknowledgements, peer review information; details of author contributions and competing interests; and statements of data and code availability are available at 10.1038/s41592-023-02086-5.

### Supplementary information

Reporting Summary

### Source data

Source Data Fig. 2 Raw data for Fig. 2a–c.

Source Data Fig. 3 Raw data for Fig. 3a.

Source Data Extended Data Fig. 2 Raw data for Extended Data Fig. a–c.

Source Data Extended Data Fig. 3 Raw data for Extended Data Fig. 3a–d.

Source Data Extended Data Fig. 4 Raw data for Extended Data Figs. a–b.

Source Data Extended Data Fig. 5 Raw data for Extended Data Fig. 5a.

## Data Availability

Source code and a link to the training weights have been made available at https://github.com/uw-ipd/RoseTTAFold2NA. Updated CASP15 RNA predictions have been made available at 10.5281/zenodo.7555957. All data used for training and evaluation is publicly available through the PDB (https://www.rcsb.org/). The data used for analyzing sequence specificity is publicly available through Cis-BP (http://cisbp.ccbr.utoronto.ca/). Source data are provided with this paper.

## Code availability

The code for this model is available at https://github.com/uw-ipd/RoseTTAFold2NA. This repository includes preprocessing and inference scripts, and a link to the model weights.
